# The lead-up to epidemic transmission: malaria trends and control interventions in Burundi 2000 to 2019

**DOI:** 10.1186/s12936-021-03830-y

**Published:** 2021-07-02

**Authors:** Denis Sinzinkayo, Dismas Baza, Virgile Gnanguenon, Cristian Koepfli

**Affiliations:** 1 University of Burundi, Bujumbura, Burundi; 2 National Malaria Control Program, Ministry of Health, Bujumbura, Burundi; 3 World Health Organization, Burundi Country Office, Bujumbura, Burundi; 4 PMI VectorLink Project, U.S. Agency for International Development, Abt Associates, Bujumbura, Burundi; 5 University of Notre Dame, Notre Dame, IN USA

**Keywords:** Malaria control, Outbreak, Epidemic, Intervention

## Abstract

Burundi has experienced an increase in malaria cases since 2000, reaching 843,000 cases per million inhabitants in 2019, a more than twofold increase compared to the early 2000s. Burundi thus contrasts the decreasing number of cases in many other African countries. To evaluate the impact of malaria control on this increase, data on interventions from 2000 to 2019 were compiled. Over this period, the number of health facilities increased threefold, and the number of tests 20-fold. The test positivity rate remained stable at around 50–60% in most years. Artemisinin-based combination therapy was introduced in 2003, initially using artesunate–amodiaquine and changed to artemether–lumefantrine in 2019/2020. Mass distribution campaigns of insecticide-treated bed nets were conducted, and indoor residual spraying and intermittent preventive treatment in pregnancy introduced. Thus, the increase in cases was not the result of faltering control activities. Increased testing was likely a key contributor to higher case numbers. Despite the increase in testing, the test positivity rate remined high, indicating that current case numbers might still underestimate the true burden.

## Background

Burundi is a small (27,834 km^2^) landlocked African country with historically very high *Plasmodium falciparum* malaria transmission levels. In 1990–1991, prior to any major interventions, mortality due to malaria in infants below 1 year was 38 per 1000, and 8 per 1000 in children aged 1–4 years [1]. Malaria was thus the primary cause of infant and child mortality, accounting for 30% of deaths [1], and the malaria mortality rate was amongst the highest in sub-Saharan Africa [2].

**Fig. 1:**
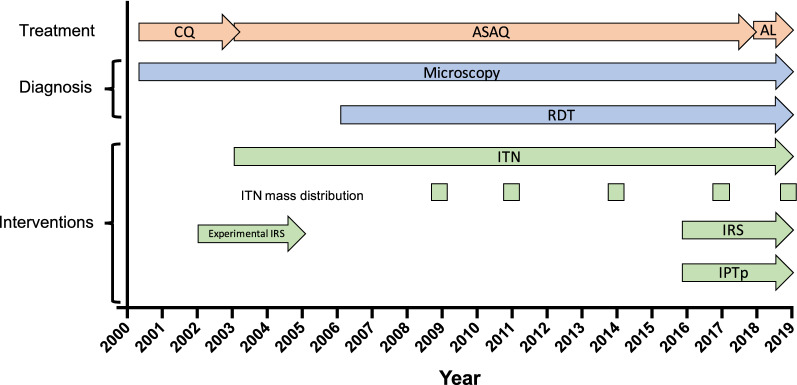
Timeline of malaria control interventions in Burundi 2000–2019. *CQ *chloroquine, *ASAQ *artesunate–amodiaquine, *AL *artemether–lumefantrine, *RDT *rapid diagnostic test, *LLIN *long-lasting insecticidal net, *IRS *indoor residual spraying, *IPTp *intermittent preventive treatment in pregnancy

Since 2000, a pronounced increase in cases has been observed, resulting in epidemic transmission starting in 2017. Over 9 million cases among the population of 11.4 million were recorded in 2019 [3]. The pattern in Burundi thus contrasts the 40% decrease in malaria cases across Africa from 2000 to 2015 [4]. This study aims to critically evaluate malaria control interventions from 2000 to 2019 (Fig. 1), and their impact on case numbers. To this aim, data on confirmed cases and deaths, and on control interventions were compiled from the Ministry of Health.

## Malaria surveillance in Burundi

Data on health care and services are collected daily at the community level, in health centres, and hospitals. Every functional health facility in Burundi has the duty to transmit data on the standard national health information systems (HIS) tools [5]. Until 2007, the data was entered and transmitted on paper to the national level where it was entered into an Excel database. From 2008 to 2013, the health facilities had to transmit the paper report to the health district, which in turn had to record them in the GIS software. Since 2014, all the country’s public and private health facilities have been recording the data in the district health information software (DHIS2) every day. The National Health Information System Office organizes the processing and analysis of data transmitted by the health facilities through the DHIS2 in collaboration with the different programs and health departments. It evaluates the completeness, timeliness and quality of the data and then prepares periodic reports, including the statistical yearbook and bulletins. It also provides feedback to the health districts which in turn provide feedback to the health facilities.

Possible poor quality and lack of completeness of data is a limitation of this report and hampers surveillance in Burundi. According to the National Health Information System Data Quality Improvement Strategic Plan 2019–2023, in 2018, the overall completeness of health facility reports was 92%. Only one health district completed < 80% of reports. None of the monthly reports from the health zones had zero/missing values [5]. Over the year 2017, the number of confirmed malaria cases in 10 health districts show a deviation of more than 33% from expected values, possibly indicating missing data [5].

## Case numbers and testing

**Fig. 2:**
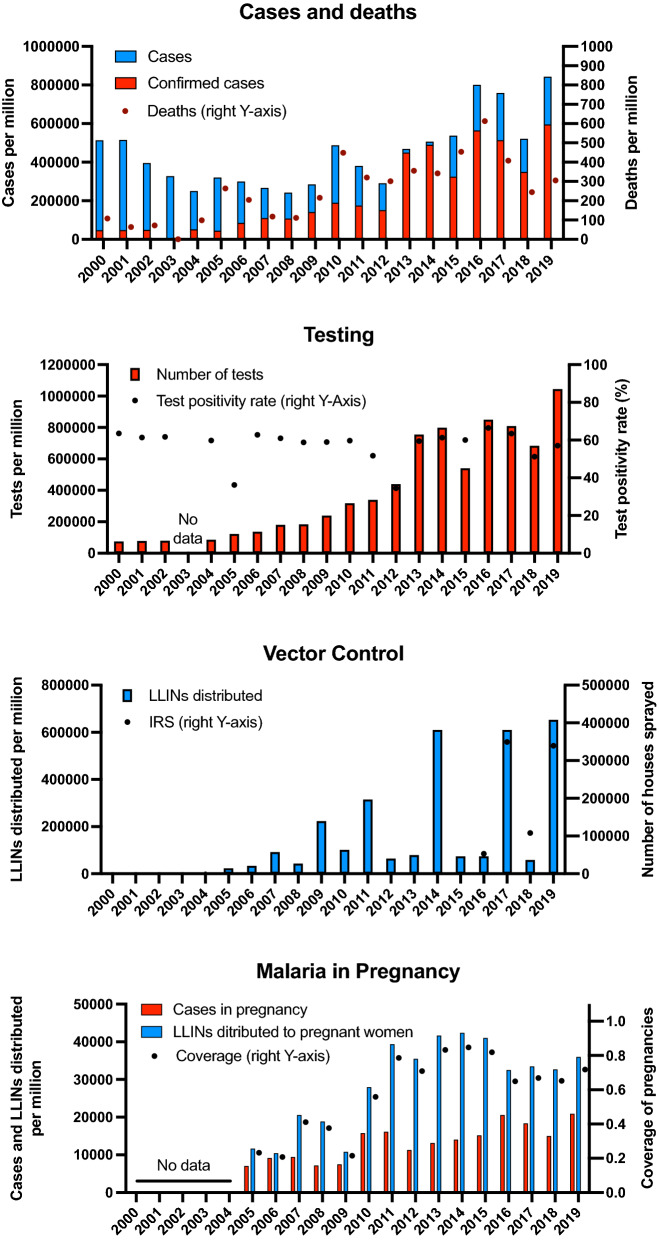
Malaria burden and intervention coverage in Burundi 2000–2019. **A** cases and deaths per million inhabitants per year, **B** number of tests per million inhabitants per year, **C** LLINs distributed per million inhabitants per year, and number of households sprayed, **D** malaria cases in pregnancy. Numbers are given per million inhabitants per year, as the number of pregnancies was not available. *LLIN *long-lasting insecticidal net, *IRS *indoor residual spraying

From 2000 to 2019, the population of Burundi almost doubled from 6.5 to 11.5 million. In the same period, the number of *P. falciparum* cases increased threefold from 3,272,608 to 9,416,989. Thus, the cases per million population increased from 513,000 to 843,000. The number of confirmed deaths per year per million increased from 60 to 110 in 2000–2004 to 300–600 in more recent years (Fig. 2A). The case definition of malaria follows World Health Organization (WHO) recommendations for suspected and confirmed cases [6] and did not change over the study period.

Testing capacity, and the number of tests conducted, grew over time. While in 2000 only 494 health centres and hospitals had the capacity to diagnose malaria, this number increased to 1359 until 2019. The number of tests increased from less than half a million in 2000 to over 12 million in 2019. On a per-capita basis, the number of tests increased more than 12-fold, from under 75,000 per million in 2000 to 1,044,000 in 2019 (Fig. 2B).

The proportion of confirmed cases increased from 9.2% to 2000 to 70.6% in 2019 (Fig. 2A). From 2000 to 2019, with few exceptions, the test positivity rate remained relatively stable at 52–65% (Fig. 2B). Only in 2005 (36.2%) and 2012 (34.5%) it was lower. A decline in the positivity rate in response to a strong increase in testing could indicate that an increasing proportion of all cases are detected. The consistently high positivity rate despite increased testing indicates that the number of tests is still too small, and that case numbers are still underestimated.

Until 2005, all testing was done by light microscopy. In 2006, rapid diagnostic tests (RDTs) were introduced, and by 2018 were used for over 75% of malaria diagnosis. Across all years, test positivity rates by RDT and microscopy were nearly identical (RDT: 56.0%, microscopy: 56.7%), suggesting similar diagnostic sensitivity of the two methods. Different brands of RDT were used in Burundi, as funders made their own choices under the condition that the RDT is prequalified by the WHO. All health providers at public and private health centres are trained in RDT and microscopy use. The national malaria control program organizes quarterly formative supervision on laboratory test quality assurance targeting the private and public sector.

## Treatment

From 2003 to 2019, artesunate-amodiaquine (ASAQ) was used as first-line treatment. Artemisinin therapeutic efficacy trials have been conducted in Burundi three times. In 2002 the treatment response rate for ASAQ and artemether–lumefantrine (AL) was > 95% and > 99% respectively [7]. Artesunate–amodiaquine has been chosen as first-line treatment as it was more economical. In 2006, the adequate treatment response rate for ASAQ was 94%; and in 2019 it was 92%. In 2020, Burundi introduced AL as first line treatment for uncomplicated malaria to harmonize treatment policy within East Africa Community countries along with Rwanda and Tanzania. Furthermore, patients’ adherence to treatment regimens with ASAQ had been a persistent issue; they preferred purchasing in the private sector instead of taking ASAQ for free in public health centres [8].

## Vector control

Distribution of long-lasting insecticidal nets (LLINs) started in 2003 through social marketing campaigns. Since 2005, distribution of LLINs were integrated with other routine health centre-based services (antenatal care visits and measles vaccination of children). LLINs mass distributions campaigns in Burundi were initiated for the first time in 2009, targeting the most vulnerable people (children under 5 and pregnant women). Distribution was integrated to measles vaccination campaigns; 1.6 million LLINs were distributed among the 8.4 million inhabitants. Further mass distribution campaigns took place in 2011, 2014, 2017, and 2019 (Fig. 1D). In the latter three campaigns, within the framework of universal access to malaria preventive interventions, one bed net per two persons was distributed in line with WHO standards.

All LLINs distributed until 2018 were permethrin-based LLINs (Olyset Net®), alphacypermethrin-based LLINs (Royal Sentry®), and deltamethrin-based LLINs (Netprotect®, and Permanet®). In 2019, pyrethroid-PBO LLINs were distributed in 4 health districts with identified *Anopheles* resistance to pyrethroid. All pyrethroid-only nets were removed from these health districts, and PBO nets are distributed through mass distributions campaigns and routine health centre-based services (antenatal care visits and measles vaccination of children). Deltamethrin-based LLINs (Yorkool®) were distributed in all the remaining districts.

The LLIN coverage after the 2014 mass-distribution campaign was estimated at 97%, and after the December 2019 campaign at 96.7%. However, the durability monitoring early findings demonstrated that the LLINs survivorship in the households was 63% for the standard LLINs and 88% for the PBO LLINs 8 months after the mass-distribution campaign [9]. This suggests a rapid loss of coverage.

The campaigns in 2011 and 2017 might have resulted in a reduction in case numbers. From 2011 to 2012, the number of tests increased from 340,000 to 439,000 per million, yet case numbers decreased from 380,000 to 290,000 per million. Test positivity rate in 2012 was the lowest over the period studied at 34.5%. From 2017 to 2018, the number of tests decreased from 810,000 to 684,000 per million. Case numbers declined from 759,000 to 522,000 per million, resulting in a moderate decrease of the test positivity rate from 63 to 51%. After the 2014 campaign, the number of tests decreased, but the number of cases increased moderately (Fig. 2A, C).

A major outbreak in the northern part of the country was recorded in 2000–2001 [10, 11]. In response, indoor residual spraying (IRS) in combination with distribution of ITNs was conducted in Karuzi in the highlands [12]. High coverage of IRS was achieved (over 99% of houses sprayed), and vector density decreased significantly. Prevalence of infection by RDT did not decrease in zones with IRS, but was lower in individuals sleeping under an ITN [12].

Following the intervention in 2000–2001, IRS in combination with bed net distributions were trialed further in Karuzi in 2002–2005. Interventions were conducted within a horizontal range of 700 m from the bottom of the valleys, were transmission was highest. In the frame of the trial, 24,000 bed nets were distributed. IRS was conducted once per year for 4 consecutive years, each time between 14,000 and 18,000 houses were sprayed [13]. Vector density and the number of infective bites were reduced 80–90% [13]. Infection prevalence and the risk of clinical malaria were reduced by 40–50% [14]. Despite the notion that transmission in the areas at higher altitudes might be fueled by vectors migrating from the valleys, no impact was observed in these zones were no interventions took place.

Despite the positive outcome of the trial in Karuzi, not further IRS was conducted for a decade. Regular IRS started in 2016 and is conducted annually in 4/47 health districts with high case numbers. Additional Health districts are being sprayed occasionally in response to malaria outbreaks. Over 300,000 structures were sprayed in 2017 and 2019 (Fig. 2C, one household typically consists of around three structures). Due to the responsive nature of the IRS and the focus on only a few sites, current IRS activities are insufficient to achieve a reduction of case numbers on the country level.

## Malaria in pregnancy

Since 2011, between 600,000 and 750,000 bed nets are distributed each year during antenatal care visits and delivery. Since 2010, 60–80% of targeted pregnant women receive a bed bet (Fig. 1D). Intermittent preventive treatment in pregnancy (IPTp) started in 2016. The number of malaria cases in pregnancy increases until 2016 and since remained stable. Other indicators such as maternal anemia or birth weight or the number of preterm births are not available.

## Current and future challenges

The rapid population growth remains a challenge for any intervention. Based on numbers from the World Bank, with a fertility rate of 5.4 births per women, Burundi is among the 10 fastest growing nations on the planet [15]. A steady increase of resources for malaria control is required to maintain the level of protection, and even more resources to expand control.

From 1994 to 2015, the country experienced repetitive political crises which caused movements of the population to internal refugee camps and to neighboring countries. Migration intensified at the time of presidential elections (2005, 2010, 2015, 2020). According to the UNHCR, from 2015 to 2017, over 420,000 Burundians have left the country, and a further 55,000 were displaced within the country [16]. Further displacements occurred in recent years [17]. Often displaced populations are not sufficiently protected against malaria [18, 19]. Changes in land use are likely another factor adding to conditions favoring transmission. In the last 10 years, rice plantations were expanded in many mountainous regions of Burundi, creating larval habitats [20, 21].

Resistance has been documented against all available antimalarial drugs, and is a constant threat to malaria control. Emergence of indigenous resistance to the first-line drug AL has been documented in Equatorial Guinea [22], and mutations associated with resistance have been reported from neighbouring Rwanda [23, 24]. Emergence of de-novo drug resistance, or importation of mutant parasites is a serious threat. A new therapeutic efficacy study is planned for 2021.

The use of RDTs is threatened by *hrp2* deletions, resulting in false-negative tests. Deletions have been reported in multiple countries in Africa, reaching very high frequency at the Horn of Africa [25]. Molecular monitoring of *hrp2* deletion is a high-priority task of the WHO [26], and is particularly relevant in Burundi as deletions have been reported in neighbouring Democratic Republic of Congo (DRC) [27].

The success of LLIN distribution campaigns and IRS, which was recently scaled up in Burundi, crucially depends on killing of mosquitos by the insecticides used. Growing levels of insecticide resistance are a thread throughout Africa [28]. In response to the spraying campaigns in the highlands in 2002–2005 [13], levels of *kdr* mutations increased from 1 to 86% [29]. New studies on insecticide resistance have been started in 2017 (Gnanguenon et al., in preparation).

Burundi did well in containing the spread of SARS-CoV-2 in the first months of the pandemic. Border closures were the most important intervention. Only 818 confirmed cases were reported until December 31st, 2020. Since then, cases numbers have increased to over 4700 as of May 31, 2021. The pandemic did not interrupt malaria control interventions, i.e. IRS and LLIN distribution. Vector control and case management interventions were adapted as per WHO guidelines to mitigate the impact of covid-19 on delivery of HIV, tuberculosis, and malaria control services [30]. The pandemic has, however, affected the supply chain of malaria drugs at health posts in the communities, and some surveillance activities. The change from ASAQ to AL was planned to be completed nationwide in mid-2020, but delayed because insufficient amounts of AL were received. It was also planned to introduce artesunate suppositories in the community health posts for severe cases before they are transferred to health centres or hospitals. Due to delays in receiving the drugs, currently only 17 out of 27 health districts have resumed malaria treatment at community health posts. Lastly, the LLIN durability study was delayed by 1 to 2 months because of COVID-19 [31].

## Conclusions

The steadily increasing number of malaria cases in Burundi since 2000 was observed despite a large increase in the number of health facilities, number of tests conducted, number of bed nets distributed, changes in treatment guidelines, and more recently introduction of IRS and IPTp. Despite a 20-fold increase in testing, the test positivity rate remained stable at around 50–60%. Given low testing numbers in the early phase of the study period, case numbers were likely underestimated. The consistently high positivity rate indicates that still many cases are missed.

In order to reduce the burden of malaria in Burundi, existing control activities need to be scaled up. New interventions that are currently tested in different settings might aide malaria control in the future, e.g. novel vector control tools such as larviciding [32], or activities to target the asymptomatic reservoir, such as chemoprevention in children [33]. In parallel continued surveillance of drug efficacy, insecticide resistance, and *hrp2* deletion is recommended.

## Data Availability

All data generated or analyzed during this study are included in this published article.
